# The comparison of post-treatment quality of life after at least two years of follow-up (≥ 24 months) between robotic radical prostatectomy and radiotherapy for intermediate-risk localized prostate cancer

**DOI:** 10.1186/s12894-025-02018-9

**Published:** 2026-02-05

**Authors:** Selahattin Bedir, Burak Ünal, Turgay Ebiloglu, Selçuk Demiral, Selçuk Sarıkaya, Mehmet Murat Beyzadeoğlu

**Affiliations:** 1 Department of Urology, Gülhane Training and Research Hospital, Etlik, Ankara, Turkey; 2 Department of Radiation Oncology, Gülhane Training and Research Hospital, Etlik, Ankara, Turkey

**Keywords:** Quality of life, Prostate cancer, Robotic radical prostatectomy, Radiotherapy, IMRT

## Abstract

**Background:**

Robot-assisted laparoscopic radical prostatectomy (RALRP) and intensity-modulated radiotherapy (IMRT) are the main curative options for localized prostate cancer (PCA). However, both may significantly affect patients’ quality of life (QoL).

**Objective:**

To retrospectively compare post-treatment QoL after at least 24 months of follow-up between bladder-neck- and nerve-sparing RALRP and IMRT in patients with intermediate-risk localized PCA.

**Methods:**

A total of 164 patients treated between October 2016 and May 2023 were analyzed (104 RALRP; 60 IMRT). QoL was evaluated using the Expanded Prostate Cancer Index Composite (EPIC-2002). Urinary, bowel, sexual, and hormonal domains were compared using appropriate statistical tests .

**Results:**

Baseline demographics were similar between groups. The mean age was 68.82 ± 6.72 years in the RALRP group and 68.89 ± 7.81 years in the IMRT group. IMRT patients reported better sexual function (*p* < 0.05), but this advantage was counteracted by hormonal side effects due to androgen deprivation therapy (ADT). Urinary continence was comparable (*p* > 0.05), whereas irritative urinary symptoms were more common in IMRT (*p* < 0.001). Bowel function and overall satisfaction were significantly better in RALRP (both *p* < 0.001).

**Conclusions:**

After ≥ 24 months of follow-up, RALRP provided superior bowel and hormonal outcomes, while IMRT yielded slightly better sexual function scores. Overall satisfaction favored RALRP.

## Introduction

Prostate cancer (PCA) is the most frequently diagnosed malignancy among men and the second leading cause of cancer-related death worldwide [1].

Despite advances in screening and treatment, both radical prostatectomy and radiotherapy remain standard curative options [2, 3].

The development of robot-assisted laparoscopic radical prostatectomy (RALRP) and intensity-modulated radiotherapy (IMRT) has improved oncologic control and functional outcomes [4–6].

However, these modalities can have distinct effects on post-treatment quality of life (QoL) [7–9].

RALRP offers enhanced visualization and neurovascular preservation, while IMRT allows precise dose delivery with minimized toxicity [10].

The European Association of Urology (EAU) 2025 guidelines [11] emphasize individualized treatment selection, yet real-world QoL data remain limited.

Thus, we aimed to compare post-treatment QoL after ≥ 24 months between RALRP and IMRT in intermediate-risk localized PCA.

## Materials and methods

### Study design and ethical approval

This retrospective study was approved by the Institutional Review Board of Gülhane Training and Research Hospital (approval no: 2023 − 356).

All procedures adhered to the Declaration of Helsinki. Consent to participate: Not applicable. This study was retrospective, and all data were collected from existing medical records. Clinical trial number: Not applicable. Ethics approval and consent to participate: The study was approved by the Institutional Ethics Committee of Gülhane Training and Research Hospital (approval number: 2023 − 356). Consent for publication: Not applicable.

### Patient selection

Between October 2016 and May 2023, 875 men received definitive treatment for localized PCA: 500 underwent RALRP and 375 IMRT.

From these, 164 intermediate-risk patients (per EAU 2025 criteria [11]) with ≥ 24 months of follow-up were included (104 RALRP, 60 IMRT).

Exclusion criteria: high- or low-risk disease, prior pelvic therapy, incomplete Expanded Prostate Cancer Index Composite (EPIC-2002) data, or follow-up < 24 months.

### Treatment protocols

RALRP was performed using a bladder-neck- and nerve-sparing approach [12, 13].

Postoperatively, patients received 5 mg tadalafil daily for 12 months and performed Kegel exercises.

IMRT was delivered as image-guided 3D radiotherapy (78–80 Gy total) [10].

All IMRT patients received 6 months of androgen deprivation therapy (ADT), per EAU guidelines [11].

### Pre-treatment evaluation

Screening included prostate-specific antigen (PSA), digital rectal examination (DRE), multiparametric magnetic resonance imaging (mpMRI) using the Prostate Imaging Reporting and Data System (PI-RADS v2.1), and cognitive fusion biopsy [11].

Gleason scores and PSA density were recorded, and staging (Ga-68 PSMA PET/CT or CT) excluded metastasis.

### Quality-of-life assessment

QoL was evaluated using the EPIC-2002 at ≥ 24 months post-treatment [7].

Domains included urinary, bowel, sexual, hormonal, and overall satisfaction.

### Statistical analysis

Sample size (G*Power 3.1) indicated 104 subjects per group for 90% power (α = 0.05).

Data were analyzed with SPSS 26.0.

Continuous variables: t-test or Mann–Whitney U.

Categorical: chi-square.

Significance: *p* < 0.05.

## Results

### Baseline characteristics

The mean age was 68.82 ± 6.72 years in the RALRP group and 68.89 ± 7.81 years in the IMRT group (*p* = 0.952). There were no significant differences in age, PSA, PSA density, prostate volume, or Gleason scores between the groups (all *p* > 0.05).

(Table 1)

**Table 1:** Patients’ characteristics

	G1(RALRP)	G2(IMRT)	*p*
Age	68.82±6.72 (43–83)	68.89±7.81 (57–90)	0.952
Median and mean PSA (ng/ml)	7.75 and 6.08±6.82 (2.5–17.8.5.8)	8.86 and 5.68±9.1 (2.5–19.2.5.2)	0.187
The median and mean free/total PSA ratio	11% and 13%±7% (0–44%)	10% and 15%±7% (3–55%)	0.344
The Prostate MRI	2 (1%) PIRADS 1 15 (15%) PIRADS 2 17 (16%) PIRADS 3 46 (44%) PIRADS 4 24 (22%) PIRADS 5	3 (4%) PIRADS 2 29 (48%) PIRADS 4 28 (48%) PIRADS 5	0.102
Prostate volume(ml)	68±54 (24–123)	73±43 (29–132)	0.812
The median and mean PSAD	0.20 and 0.27±0.28 (0.08–2.12.08.12)	0.34 and 0.4±0.29 (0.01–1.01)	0.098
Pathologic results of biopsy	65 (62%) gleason 3+3 23 (22%) gleason 3+4 16 (16%) gleason 4+3	21 (35%) gleason 3+3 13 (22%) gleason 3+4 26 (43%) gleason 4+3	0.513

*Abbreviations*: *PSA* Prostate-specific antigen, *PSAD* Prostate-specific antigen density

### Urinary function

Urinary incontinence was similar (*p* = 0.691).

Irritative symptoms (dysuria, frequency) were higher in IMRT (*p* < 0.001).

RALRP patients had better urinary control and pad use (*p* < 0.001).

Overall urinary satisfaction favored RALRP (*p* < 0.001).

(Table 2)

**Table 2:** The comparison for urinary function

			G1(RALRP), %	G2(IMRT), %	*P*
*Urinary problems*					
Over the past 4 weeks, how often have you leaked urine?		Rarely or never	32	36	0.691
		About once a week	11	7	
		More than once a week	19	18	
		About once a day	17	11	
		More than once a day	20	27	
Over the past 4 weeks, how often have you urinated blood		Rarely or never	90	95	0.528
		About once a week	0	0	
		More than once a week	7.9	2.4	
		About once a day	1.3	0	
		More than once a day	1.3	2.4	
Over the past 4 weeks, how often have you had pain or burning with urination?		Rarely or never	83	52	<0.001
		About once a week	5.3	14	
		More than once a week	10.5	11.6	
		About once a day	1.3	4.7	
		More than once a day	0	20	
Which of the following best describes your urinary control during the last 4 weeks?		No urinary control whatsoever	6.6	12.2	0.001
		Frequent dribbling	27.6	22	
		Occasional dribbling	5.3	29.3	
		Total control	60.5	34.1	
How many pads or adult diapers per day did you usually use to control leakage during the last 4 weeks?		None	50	56	<0.001
		1 pad per day	1.3	16.3	
		2 pads per day	17	4.7	
		3 or more pads per day	6.6	18.6	
How big a problem, if any, has each of the following been for you during the last 4 weeks?	Dripping or leaking urine	No problem	60	28	<0.001
		Very small problem	2.6	22.2	
		Small Problem	5.3	11.1	
		Modarate problem	27.6	19.4	
		Big problem	5.3	19.4	
	Pain or burning on urination	No problem	61	42	0.010
		Very small problem	13.2	8.6	
		Small Problem	21.1	28.6	
		Modarate problem	1.3	17.1	
		Big problem	2.6	0	
	Bleeding with urination	No problem	85	86	0.091
		Very small problem	0	2.8	
		Small Problem	14.5	5.6	
		Modarate problem	0	2.8	
		Big problem	0	2.8	
	Weak urine stream or incomplete emptying	No problem	60	27	<0.001
		Very small problem	11.8	13.5	
		Small Problem	2.6	24.3	
		Modarate problem	22.4	24.3	
		Big problem	2.6	10.8	
	Waking up to urinate	No problem	1.3	27.9	<0.001
		Very small problem	61.8	12.5	
		Small Problem	11.8	15	
		Modarate problem	22.4	37.5	
		Big problem	2.6	7.5	
Overall, how big a problem has your urinary function been for you during the last 4 weeks?		No problem	1.3	26.2	<0.001
		Very small problem	71.1	28.6	
		Small Problem	3.9	19	
		Modarate problem	22.4	19	
		Big problem	1.3	7.1	

*Abbreviations*: *PSA* Prostate-specific antigen, *PSAD* Prostate-specific antigen density

### Bowel function

Bowel symptoms (urgency, leakage, rectal pain) were more frequent after IMRT (*p* < 0.05).

RALRP showed better bowel satisfaction (*p* < 0.001).

(Table 3)

**Table 3:** The comparison for bowel function

			G1(RALRP), %	G2(IMRT), %	*P*
*Bowel habits*					
How often have you had rectal urgency (felt like I had to pass stool, but did not) during the last 4 weeks?		Rarely or never	82.2	65.9	<0.001
		About once a week	11.8	2.3	
		More than once a week	0	11.4	
		About once a day	0	2.3	
		More than once a day	0	18.2	
How often have you had uncontrolled leakage of stool or feces?		Rarely or never	100	77.6	0.001
		About once a week	0	11.6	
		More than once a week	0	2.3	
		About once a day	0	2.3	
		More than once a day	0	7	
How often have you had stools (bowel movements) that were loose or liquid (no form, watery, mushy) during the last 4 weeks?		Never	65.8	34.1	0.002
		Rarely	0	0	
		About half the time	34.2	63.6	
		Usually	0	2.3	
		Always	0	0	
How often have you had bloody stools during the last 4 weeks?		Never	100	95.5	0.061
		Rarely	0	4.5	
		About half the time	0	0	
		Usually	0	0	
		Always	0	0	
How often have your bowel movements been painful during the last 4 weeks?		Never	78.9	59.5	0.029
		Rarely	19.7	28.6	
		About half the time	1.3	4.8	
		Usually	0	7.1	
		Always	0	0	
How many bowel movements have you had on a typical day during the last 4 weeks?		Two or less	76.3	50	<0.001
		Three to four	22.4	17.5	
		Five or more	1.3	32.5	
How often have you had crampy pain in your abdomen, pelvis or rectum during the last 4 weeks?		Rarely or never	42.1	55.8	<0.001
		About once a week	35.5	23.3	
		More than once a week	22.4	2.3	
		About once a day	0	14	
		More than once a day	0	4.7	
How big a problem, if any, has each of the following been for you during the last 4 weeks?	Urgency to have a bowel movement	No problem	73.7	51.3	<0.001
		Very small problem	26.3	17.9	
		Small Problem	0	20.5	
		Modarate problem	0	7.7	
		Big problem	0	2.6	
	Increased frequency of bowel movements	No problem	73.7	73.7	0.02
		Very small problem	3.9	13.2	
		Small Problem	22.4	2.6	
		Modarate problem	0	7.9	
		Big problem	0	2.6	
	Watery bowel movements	No problem	53.9	72.5	0.013
		Very small problem	26.3	8.3	
		Small Problem	19.7	11.1	
		Modarate problem	0	2.8	
		Big problem	0	5.6	
	Losing control of your stools	No problem	61.8	81.6	<0.001
		Very small problem	34.2	0	
		Small Problem	3.9	5.3	
		Modarate problem	0	5.3	
		Big problem	0	7.9	
	Bloody stools	No problem	73.7	89.2	0.006
		Very small problem	22.4	2.7	
		Small Problem	3.9	0	
		Modarate problem	0	2.7	
		Big problem	0	5.4	
	Abdominal/Pelvic/Rectal pain	No problem	73.7	64.9	0.029
		Very small problem	26.3	21.6	
		Small Problem	0	2.7	
		Modarate problem	0	5.4	
		Big problem	0	5.4	
Overall, how big a problem have your bowel habits been for you during the last 4 weeks?		No problem	32.9	59.6	<0.001
		Very small problem	44.7	19	
		Small Problem	22.4	9.5	
		Modarate problem	0	9.5	
		Big problem	0	2.4	

*Abbreviations*: *PSA* Prostate-specific antigen, *PSAD* Prostate-specific antigen density

### Sexual function

Sexual function was impaired in both, slightly better in IMRT for desire and erection ability (*p* = 0.028, *p* = 0.010), consistent with prior studies.

ADT reduced libido and potency, balancing results.

(Table 4)

**Table 4:** The comparison for sexual function

				G1(RALRP), %	G2(IMRT), %	*P*
*Sexual Function*						
How would you rate each of the following during the last 4 weeks?		Your level of sexual desire?	Very poor to none	56.1	50	0.028
			Poor	19.7	23.8	
			Fair	7.9	7.1	
			Good	21.7	7.1	
			Very good	0	7.1	
		Your ability to have an erection?	Very poor to none	50	66.4	0.010
			Poor	21.1	17.9	
			Fair	10.5	10.13	
			Good	18.4	2.6	
			Very good	0	2.6	
		Your ability to reach orgasm (climax)?	Very poor to none	50	51.4	<0.001
			Poor	21.1	25.6	
			Fair	10.5	12.8	
			Good	18.4	2.6	
			Very good	0	2.6	
How would you describe the usual QUALITY of your erections during the last 4 weeks			None at all	52.6	55.2	0.228
			Not firm enough for any sexual activity	17.1	16.7	
			Firm enough for masturbation and foreplay only	9.2	11.9	
			Firm enough for intercourse	21.1	14.3	
How would you describe the FREQUENCY of your erections during the last 4 weeks?			I NEVER had an erection when I wanted one	57.9	50	0.022
			I had an erection LESS THAN HALF the time I wanted one	11.8	25	
			I had an erection ABOUT HALF the time I wanted one	11.8	10	
			I had an erection MORE THAN HALF the time I wanted one	18.4	7.5	
			I had an erection WHENEVER I wanted one	0	7.5	
How often have you awakened in the morning or night with an erection during the last 4 weeks?			Never	57	62.5	0.035
			Less than once a week	11.8	25	
			About once a week	13.2	7.5	
			Several times a week	17.1	2.5	
			Daily	0	2.5	
During the last 4 weeks, how often did you have any sexual activity?			Not at all	55.3	53.6	0.08
			Less than once a week	13.2	34.1	
			About once a week	23.7	9.8	
			Several times a week	7.9	0	
			Daily	0	2.4	
During the last 4 weeks, how often did you have sexual intercourse?			Not at all	64.5	70.7	0.085
			Less than once a week	9.2	17.1	
			About once a week	18.4	9.8	
			Several times a week	7.9	0	
			Daily	0	2.4	
Overall, how would you rate your ability to function sexually during the last 4 weeks?			Very poor	60.5	63.4	0.516
			Poor	10.5	12.2	
			Fair	21.1	22	
			Good	7.9	2.4	
			Very good	0	0	
How big a problem during the last 4 weeks, if any, has each of the following been for you?	Your level of sexual desire		No problem	7.9	10.3	0.114
			Very small problem	19.7	10.3	
			Small Problem	60.5	59	
			Modarate problem	11.8	12.8	
			Big problem	0	7.7	
	Your ability to have an erection		No problem	9.2	19.7	0.01
			Very small problem	9.6	2.6	
			Small Problem	17.1	10.3	
			Modarate problem	6.6	33.3	
			Big problem	52.6	30.8	
	Your ability to reach an orgasm.		No problem	1.3	13.5	<0.001
			Very small problem	22.4	5.4	
			Small Problem	11.8	8.1	
			Modarate problem	11.8	51.4	
			Big problem	52.6	21.6	
Overall, how big a problem has your sexual function or lack of sexual function been for you during the last 4 weeks?			No problem	9.2	25.0	<0.001
			Very small problem	15.8	5	
			Small Problem	17.1	10	
			Modarate problem	5.3	40	
			Big problem	52.6	20	

*Abbreviations*: *PSA* Prostate-specific antigen, *PSAD* Prostate-specific antigen density

### Hormonal function

IMRT patients had more fatigue, hot flashes, and mood changes due to ADT (*p* < 0.001).

RALRP had minimal hormonal complaints.

(Table 5)

**Table 5:** The comparison for hormonal function

			G1(RALRP), %	G2(IMRT), %	*P*
*Hormonal Function*					
Over the last 4 weeks, how often have you experienced hot flashes?		Rarely or never	100	59.5	<0.001
		About once a week	0	14.3	
		More than once a week	0	2.4	
		About once a day	0	7.1	
		More than once a day	0	16.7	
How often have you had breast tenderness during the last 4 weeks?		Rarely or never	100	94.6	0.124
		About once a week	0	0	
		More than once a week	0	2.7	
		About once a day	0	0	
		More than once a day	0	2.7	
During the last 4 weeks, how often have you felt depressed?		Rarely or never	98.7	73.2	0.001
		About once a week	0	12.2	
		More than once a week	1.3	9.8	
		About once a day	0	2.4	
		More than once a day	0	2.4	
During the last 4 weeks, how often have you felt a lack of energy?		Rarely or never	98.7	48.8	<0.001
		About once a week	0	20.9	
		More than once a week	1.3	4.7	
		About once a day	0	11.6	
		More than once a day	0	14	
How much change in your weight have you experienced during the last 4 weeks, if any?		Gained 10 pounds or more	0	2.4	<0.001
		Gained less than 10 pounds	0	11.9	
		No change in weight	100	66.7	
		Lost less than 10 pounds	0	16.7	
		Lost 10 pounds or more	0	2.4	
How big a problem during the last 4 weeks, if any, has each of the following been for you?	Hot flashes	No problem	100	41	<0.001
		Very small problem	0	10.3	
		Small Problem	0	28.2	
		Modarate problem	0	10.3	
		Big problem	0	10.3	
	Breast tenderness /enlargement	No problem	100	83.6	0.011
		Very small problem	0	5.4	
		Small Problem	0	5.4	
		Modarate problem	0	2.7	
		Big problem	0	2.7	
	Loss of Body Hair	No problem	100	63.9	<0.001
		Very small problem	0	25	
		Small Problem	0	0	
		Modarate problem	0	0	
		Big problem	0	11	
	Feeling depressed	No problem	86.8	67.6	0.015
		Very small problem	1.3	13.5	
		Small Problem	11.8	13.5	
		Modarate problem	0	2.7	
		Big problem	0	2.7	
	Lack of energy	No problem	86.8	62.2	<0.001
		Very small problem	1.3	5.4	
		Small Problem	11.8	8.1	
		Modarate problem	0	18.9	
		Big problem	0	5.4	
	Change in body weight	No problem	100	75.7	<0.001
		Very small problem	0	5.4	
		Small Problem	0	8.1	
		Modarate problem	0	5.4	
		Big problem	0	5.4	
Overall, how big a problem has your hormonal function been for you during the last 4 weeks?		No problem	1.3	26.2	<0.001
		Very small problem	71.1	28.6	
		Small Problem	3.9	19	
		Modarate problem	22.4	19	
		Big problem	1.3	7.1	

*Abbreviations*: *PSA* Prostate-specific antigen, *PSAD* prostate-specific antigen density

### Overall satisfaction

Higher satisfaction with RALRP (61.7% “extremely satisfied” vs. 25% in IMRT, *p* < 0.001).

(Table 6)

**Table 6:** The comparison for overall satisfaction

		G1(RALRP), %	G2(IMRT), %	*P*
*Overall satisfaction about the treatment*				
Overall, how satisfied are you with the treatment you received for your prostate cancer?	Extremely dissatisfied	2.6	4.5	<0.001
	Dissatisfied	2.6	2.3	
	Uncertain	7.9	25	
	Satisfied	19.7	43.2	
	Extremely satisfied	61.7	25	

*Abbreviations*: *PSA* Prostate-specific antigen, *PSAD* Prostate-specific antigen density

## Discussion

This study confirms both RALRP and IMRT are effective but differ in long-term QoL [7, 14, 15].

Urinary function similar; RALRP superior in bowel, hormonal, and overall satisfaction.

These findings align with Miyoshi et al. [7] and Noda et al. [15].

Sexual function slightly better in IMRT [8, 9], though ADT’s adverse effects offset this [16, 17].

Hormonal issues higher after radiotherapy, consistent with prior studies [10, 18].

Overall satisfaction favored RALRP, echoing Giberti et al. [18].

Strengths: homogeneous intermediate-risk cohort, standardized protocols, 24-month follow-up.

Limitations: retrospective design, lack of baseline QoL, smaller IMRT cohort.

## Conclusion

RALRP and IMRT both provide effective cancer control in intermediate-risk PCA.

After ≥ 24 months, RALRP offered better bowel and hormonal QoL, IMRT slightly better sexual outcomes.

Urinary function comparable.

Further multicenter prospective trials warranted.

## Data Availability

The datasets generated and/or analyzed during the current study are available from the corresponding author on reasonable request.

## Abbreviations

PCA: Prostate cancer
RALRP: Robot-assisted laparoscopic radical prostatectomy
IMRT: Intensity-modulated radiotherapy
QoL: Quality of life
PSA: Prostate-specific antigen
PSAD: Prostate-specific antigen density
DRE: Digital rectal examination
mpMRI: Multiparametric magnetic resonance imaging
ADT: Androgen deprivation therapy

## References

[1] Siegel RL, Kratzer TB, Giaquinto AN, Sung H, Jemal A. Cancer statistics, 2025. CA Cancer J Clin. 2025;75(1):10–45. 10.3322/caac.21871. 39817679 10.3322/caac.21871 PMC11745215

[2] History of cancer treatment. Am Cancer. https://www.cancer.org/content/dam/CRC/PDF/Public/6055.00.pdf. Accessed 9 Dec 2025.

[3] Hoffman RM, Koyama T, Fan KH, et al. Mortality after radical prostatectomy or external beam radiotherapy for localized prostate cancer. J Natl Cancer Inst. 2013;105(10):711–8. 10.1093/jnci/djt059. 23615689 10.1093/jnci/djt059 PMC3653822

[4] Taguchi S, Shiraishi K, Fujimura T, et al. Robot-assisted radical prostatectomy versus volumetric modulated arc therapy: comparison of front-line therapies for localized prostate cancer. Radiother Oncol. 2019;140:62–7. 10.1016/j.radonc.2019.05.015. 31176208 10.1016/j.radonc.2019.05.015

[5] Aizer AA, Yu JB, Colberg JW, McKeon AM, Decker RH, Peschel RE. Radical prostatectomy vs. intensity-modulated radiation therapy in the management of localized prostate adenocarcinoma. Radiother Oncol. 2009;93(2):185–91. 10.1016/j.radonc.2009.09.001. 19800702 10.1016/j.radonc.2009.09.001

[6] Kim YJ, Cho KH, Pyo HR, et al. Radical prostatectomy versus external beam radiotherapy for localized prostate cancer. Strahlenther Onkol. 2015;191(4):321–9. 10.1007/s00066-014-0765-3. 25339310 10.1007/s00066-014-0765-3

[7] Miyoshi Y, Morizane S, Honda M, et al. Health related quality of life in Japanese patients with localized prostate cancer: comparative retrospective study of robot-assisted laparoscopic radical prostatectomy versus radiation therapy. Yonago Acta Med. 2020;63(1):55–62. 10.33160/yam.2020.02.008. 32158334 10.33160/yam.2020.02.008 PMC7028529

[8] Donovan JL, Hamdy FC, Lane JA, et al. Patient-reported outcomes after monitoring, surgery, or radiotherapy for prostate cancer. N Engl J Med. 2016;375(15):1425–37. 10.1056/nejmoa1606221. 27626365 10.1056/NEJMoa1606221 PMC5134995

[9] Chen RC, Basak R, Meyer AM, et al. Association between choice of radical prostatectomy, external beam radiotherapy, brachytherapy, or active surveillance and patient-reported quality of life among men with localized prostate cancer. JAMA. 2017;317(11):1141–50. 10.1001/jama.2017.1652. 28324092 10.1001/jama.2017.1652 PMC6284802

[10] Cho B. Intensity-modulated radiation therapy: a review with a physics perspective. Radiat Oncol J. 2018;36(1):1–10. 10.3857/roj.2018.00122. 29621869 10.3857/roj.2018.00122 PMC5903356

[11] Cornford Tilki D, Berg RCNVder, et al. EAU Guide Line on Prostate Cancer 2025. In: Cornford P, editor., et al., 2025.

[12] Wickham JE. The new surgery. Br Méd J (Clin Res ed). 1987;295(6613):1581. 10.1136/bmj.295.6613.1581. 3121078 10.1136/bmj.295.6613.1581 PMC1257475

[13] Sukumar S, Bhandari M, Menon M. The evolution of robotic surgery and its clinical applications. In: Sukumar S, ed. Pediatric robotic and reconstructive urology a comprehensive guide. Cham: Springer; 2012.

[14] Taguchi S, Fukuhara H, Shiraishi K, et al. Radical prostatectomy versus external beam radiotherapy for cT1-4N0M0 prostate cancer: comparison of patient outcomes including mortality. PLoS One. 2015;10(10):e0141123. 10.1371/journal.pone.0141123. 26506569 10.1371/journal.pone.0141123 PMC4624690

[15] Noda M, Taguchi S, Shiraishi K, et al. Six-year outcomes of robot-assisted radical prostatectomy versus volumetric modulated arc therapy for localized prostate cancer: a propensity score-matched analysis. Strahlenther Onkol. 2024;200(8):676–83. 10.1007/s00066-023-02192-5. 38180494 10.1007/s00066-023-02192-5 PMC11272719

[16] Iwahashi Y, Wakamiya T, Kawabata H, et al. Comparison of prognosis and health-related quality of life between robot‐assisted radical prostatectomy versus high‐dose‐rate brachytherapy combined with external beam radiation therapy and hormone therapy for high‐risk prostate cancer. Prostate. 2025;85(4):327–36. 10.1002/pros.24831. 39639423 10.1002/pros.24831

[17] Nakai Y, Tanaka N, Asakawa I, et al. Quality of life in patients who underwent robot-assisted radical prostatectomy compared with those who underwent low‐dose‐rate brachytherapy. Prostate. 2023;83(7):701–12. 10.1002/pros.24507. 36879383 10.1002/pros.24507

[18] Giberti C, Gallo F, Schenone M, et al. Robotic prostatectomy versus brachytherapy for the treatment of low risk prostate cancer. Can J Urol. 2017;24(2):8728–33. 28436359

